# Cardiovascular Outcomes Across the Sodium-Glucose Cotransporter (SGLT) Selectivity Spectrum in Type 2 Diabetes and Chronic Kidney Disease: A Systematic Review of Heart Failure, Myocardial Infarction, and Stroke

**DOI:** 10.7759/cureus.117892

**Published:** 2026-10-13

**Authors:** Mbony Joshua Cho, Aakash Hans, Aftab Ali, Surksha Gul, Vishal Bhatia, Achu Fonkem

**Affiliations:** 1 Internal Medicine, Santa District Hospital, Santa, CMR; 2 Internal Medicine, White River Medical Center, Batesville, USA; 3 Pulmonary and Critical Care Medicine, The University of Texas Medical Branch at Galveston, Galveston, USA; 4 Research, Henry Ford Health, Detroit, USA; 5 Internal Medicine, Chandka Medical College, Larkana, PAK; 6 Internal Medicine, Ghulam Muhammad Mahar Medical College, Sukkur, PAK; 7 Internal Medicine, Khairpur Medical College, Khairpur, PAK; 8 Internal Medicine, Allama Iqbal Medical College, Lahore, PAK

**Keywords:** canagliflozin, cardiovascular outcomes, chronic kidney disease, heart failure, myocardial infarction, sglt1 inhibition, sglt2 inhibitors, sotagliflozin, stroke, type 2 diabetes mellitus

## Abstract

Adults with type 2 diabetes (T2D) mellitus and chronic kidney disease (CKD) face substantial risks of heart failure and atherothrombotic cardiovascular events. Although sodium-glucose cotransporter (SGLT) inhibitors are commonly considered a single therapeutic class, differences in SGLT2 and SGLT1 selectivity may influence their cardiovascular outcome profiles. This systematic review examined whether protection against myocardial infarction, stroke, and heart failure varied across highly selective SGLT2 inhibitors, relatively less selective canagliflozin, and dual SGLT1/2 inhibition with sotagliflozin. MEDLINE through PubMed, Embase, Cochrane CENTRAL, and the Web of Science Core Collection were searched from inception to July 1, 2026, in accordance with the Preferred Reporting Items for Systematic Reviews and Meta-Analyses 2020 statement. Randomized, placebo-controlled trials enrolling adults with T2D and CKD, or providing separately extractable results for this population, were eligible. Seven parent trials, represented by 16 linked reports, were included. Their overall populations collectively comprised 61,857 participants, although eligible evidence was derived from either complete trial populations or extractable T2D-CKD subgroups. Narrative synthesis was undertaken because of heterogeneity in CKD definitions, baseline cardiovascular risk, outcome reporting, and subgroup availability. Protection against heart failure-related outcomes was observed consistently across the pharmacological spectrum. In SCORED, sotagliflozin provided the clearest concurrent signal for reductions in total major adverse cardiovascular events, myocardial infarction, and stroke. Canagliflozin reduced major cardiovascular events and hospitalization for heart failure, but its effects on individual myocardial infarction and stroke outcomes were statistically inconclusive. Highly selective SGLT2 inhibitors predominantly demonstrated heart failure and cardiorenal benefits, while CKD-specific estimates for individual atherothrombotic outcomes were frequently unavailable or inconclusive. No parent trial was judged to have a high overall risk of bias, although reliance on secondary and post hoc analyses introduced some concerns. Greater SGLT1 engagement may plausibly broaden cardiovascular protection beyond the established heart failure benefit of SGLT inhibition; however, receptor selectivity was not randomized, and cross-trial differences may reflect variation in populations, kidney phenotypes, endpoints, follow-up, and statistical power. Direct comparative or individual-participant-data analyses are required before differential cardiovascular effects can be attributed to pharmacological selectivity.

## Introduction and background

Type 2 diabetes (T2D) mellitus and chronic kidney disease (CKD) frequently coexist and together confer substantial risks of cardiovascular morbidity and premature mortality. This excess risk is not attributable to a single disease pathway. Reduced estimated glomerular filtration rate (eGFR), albuminuria, endothelial dysfunction, vascular inflammation, arterial stiffness, and altered volume homeostasis contribute to both atherothrombotic events and heart failure [1]. Myocardial infarction and stroke predominantly reflect vascular and thrombotic processes, whereas hospitalization for heart failure is more closely associated with congestion, ventricular dysfunction, and cardiorenal interactions. The relative contribution of these pathways varies with kidney disease severity, albuminuria, established atherosclerotic cardiovascular disease (ASCVD), and pre-existing heart failure, making adults with diabetes and CKD a clinically heterogeneous population [2].

Sodium-glucose cotransporter (SGLT) inhibitors have become central to the management of this high-risk population. Large cardiovascular and kidney outcome trials have demonstrated that SGLT2 inhibition can reduce heart failure events and slow CKD progression, with benefits that extend beyond glucose lowering. Proposed mechanisms include restoration of tubuloglomerular feedback, natriuresis, osmotic diuresis, reduction of interstitial congestion, preservation of kidney function, and potential improvements in myocardial energetics and inflammatory signaling [3]. Nevertheless, the effects of these agents appear less uniform for myocardial infarction and stroke than for heart failure outcomes. This distinction is clinically important because reductions in a composite cardiovascular endpoint may conceal meaningful variation among its atherothrombotic, heart failure, and mortality components.

Although commonly considered a single therapeutic class, SGLT inhibitors differ in their relative activity at SGLT2 and SGLT1. Empagliflozin, dapagliflozin, and ertugliflozin are highly selective for SGLT2, whereas canagliflozin has comparatively lower SGLT2 selectivity and may represent an intermediate pharmacological phenotype. Sotagliflozin inhibits both SGLT2 and SGLT1, including intestinal SGLT1, which contributes to postprandial glucose absorption [4]. Greater SGLT1 engagement could plausibly influence glycemic variability and other metabolic or vascular pathways, but whether these pharmacological differences translate into distinct cardiovascular outcome profiles remains uncertain. Receptor selectivity is continuous and concentration-dependent, and different degrees of selectivity have not been evaluated in direct randomized comparisons. Consequently, apparent differences between trials may reflect variation in enrolled populations, kidney phenotypes, follow-up, background therapy, endpoint definitions, or statistical power rather than receptor selectivity itself [5].

This systematic review aimed to examine whether the pattern of cardiovascular protection in adults with T2D and CKD differed across a pharmacological spectrum ranging from highly selective SGLT2 inhibition, through intermediate-selectivity canagliflozin, to dual SGLT1/2 inhibition with sotagliflozin. Particular attention was given to myocardial infarction, stroke, hospitalization for heart failure, major adverse cardiovascular events (MACE), and cardiovascular death, as well as to the potential modifying roles of eGFR, albuminuria, established ASCVD, and pre-existing heart failure. Rather than presuming comparative superiority, the review sought to identify clinically meaningful patterns, clarify the limitations of indirect cross-trial interpretation, and define priorities for future comparative and mechanistic research.

## Review

Materials and methods

Study Design and Reporting Framework

This systematic review was designed to evaluate whether the cardiovascular outcome profile of SGLT inhibition in adults with T2D mellitus and CKD varied according to pharmacological selectivity, ranging from highly selective SGLT2 inhibition, through the relatively lower selectivity of canagliflozin, to dual SGLT1/2 inhibition with sotagliflozin. The review was conducted and reported in accordance with the Preferred Reporting Items for Systematic Reviews and Meta-Analyses (PRISMA) 2020 statement [6]. The processes of record identification, deduplication, screening, eligibility assessment, and final inclusion were documented using a PRISMA 2020 flow diagram. A review protocol specifying the eligibility criteria, outcomes, and planned methods of synthesis was developed before formal screening commenced.

Review Question and PICO Framework

The review question was structured according to the population, intervention, comparator, and outcomes (PICO) framework. The population comprised adults with T2D and CKD, either constituting the complete randomized population or an independently extractable subgroup of a larger cardiovascular or kidney outcome trial. CKD was accepted when defined by reduced eGFR, increased urinary albumin-to-creatinine ratio (UACR), investigator-diagnosed diabetic kidney disease, or a combination of these criteria. Eligible interventions were classified along a pharmacological selectivity spectrum comprising the highly selective SGLT2 inhibitors empagliflozin, dapagliflozin, and ertugliflozin; canagliflozin as an intermediate or relatively less selective SGLT2 inhibitor; and sotagliflozin as a dual SGLT1/2 inhibitor. The comparator was placebo administered in addition to usual or guideline-directed care. Outcomes of interest included MACE, myocardial infarction (MI), stroke, hospitalization for heart failure (HHF), urgent visits for heart failure (HF), cardiovascular death, and composites incorporating these events. The principal comparison concerned the balance of HF-related and atherothrombotic protection across the selectivity spectrum rather than estimation of a conventional head-to-head treatment effect.

Information Sources and Search Period

MEDLINE through PubMed, Embase, the Cochrane Central Register of Controlled Trials, and the Web of Science Core Collection were searched from database inception to July 1, 2026. ClinicalTrials.gov and the World Health Organization International Clinical Trials Registry Platform were consulted to verify trial registration details and identify associated protocols and publications. Reference lists of included reports, relevant systematic reviews, and major cardiovascular and kidney outcome-trial publications were also screened manually. No publication-date restriction was applied before the final search date, and no restrictions based on country or clinical setting were imposed. Publications in languages that could not be reliably translated were documented during full-text assessment rather than excluded during title and abstract screening.

Search Strategy

The search strategy combined controlled vocabulary and free-text terms relating to T2D, CKD, SGLT inhibition, cardiovascular outcomes, and randomized trials. Terms within each concept were combined using the Boolean operator “OR,” while the principal concepts were connected using “AND.” Diabetes-related terms included “type 2 diabetes,” “type 2 diabetes mellitus,” “T2DM,” and “diabetes mellitus, type 2.” Kidney-related terms included “chronic kidney disease,” “CKD,” “diabetic kidney disease,” “diabetic nephropathy,” “renal impairment,” “reduced eGFR,” “estimated glomerular filtration rate,” “albuminuria,” and “urinary albumin-to-creatinine ratio.” Intervention terms included “SGLT inhibitor,” “SGLT2 inhibitor,” “sodium-glucose cotransporter 2 inhibitor,” “dual SGLT1/2 inhibitor,” “empagliflozin,” “dapagliflozin,” “ertugliflozin,” “canagliflozin,” and “sotagliflozin.” Cardiovascular terms included “cardiovascular outcome,” “major adverse cardiovascular event,” “MACE,” “myocardial infarction,” “stroke,” “heart failure,” “hospitalization for heart failure,” “urgent heart failure visit,” and “cardiovascular death.” Study-design terms included “randomized,” “randomized,” “randomized controlled trial,” “clinical trial,” and “placebo,” with truncation applied where appropriate, such as “random*” and “trial*.” MeSH terms in PubMed and corresponding Emtree terms in Embase were combined with terms searched in title, abstract, and keyword fields. The publication-date field was restricted from database inception through 1 July 2026, which represented the final search date. Boolean operators, proximity operators, truncation, phrase searching, and field restrictions were adapted to the requirements of PubMed, Embase, CENTRAL, and Web of Science. Searches were not restricted to records explicitly labelled as CKD subgroup analyses because eligible kidney-specific cardiovascular results could appear in secondary reports linked to broader parent trials.

Eligibility Criteria

Randomized, placebo-controlled cardiovascular or kidney outcome trials were eligible when they evaluated an SGLT inhibitor and provided clinically adjudicated cardiovascular outcomes for adults with both T2D and CKD. Trials enrolling an exclusively T2D-CKD population were included directly. Broader T2D cardiovascular outcome trials were eligible when outcomes for participants with CKD could be extracted using prespecified or reported eGFR and albuminuria criteria. Similarly, trials enrolling patients with CKD irrespective of diabetes status were eligible when results for the T2D subgroup were separately available. Parent trial reports and linked prespecified, secondary, exploratory, or post hoc analyses were eligible when they contributed nonduplicative information regarding CKD definitions, baseline cardiovascular risk, MI, stroke, HHF, cardiovascular death, or MACE.

Nonrandomized studies, observational cohorts, case-control studies, cross-sectional studies, single-arm trials, case reports, narrative reviews, systematic reviews, meta-analyses, editorials, conference summaries without sufficient outcome data, and pharmacokinetic or mechanistic studies without clinical cardiovascular outcomes were excluded. Trials restricted to type 1 diabetes, pediatric populations, pregnancy, kidney transplantation, dialysis, acute myocardial infarction, or acute HF were excluded unless a relevant T2D-CKD population and the outcomes required for this review were separately extractable. Studies reporting only glycemic, hemodynamic, imaging, functional, or kidney surrogate outcomes without MACE, MI, stroke, HHF, or cardiovascular death were also excluded. Active-comparator studies without a placebo or usual-care control were not used as primary evidence because they were not directly comparable with the selected outcome trials.

Study Selection and Management of Linked Reports

All retrieved records were combined and duplicates were removed before screening. Two reviewers independently screened titles and abstracts against the prespecified eligibility criteria, after which potentially relevant articles underwent independent full-text assessment. Disagreements were resolved through discussion and consensus, with consultation of a third reviewer when agreement could not be reached. Reasons for full-text exclusion were recorded and reported in the PRISMA flow diagram. Trial names, registration numbers, study populations, recruitment periods, intervention arms, and author groups were compared to identify multiple publications arising from the same randomized population. The parent trial, rather than the individual publication, was treated as the unit of inclusion. Secondary reports were linked to their corresponding parent trials and used only to supplement outcome-specific or CKD-specific information, thereby preventing double counting of participants.

Data Extraction and Outcomes

Two reviewers independently extracted data using a standardized form, with discrepancies resolved by consensus. Extracted variables included the parent trial and contributing reports, publication year, sample size, CKD definition, baseline eGFR and UACR, prevalence of ASCVD and HF, intervention and dose, comparator, pharmacological selectivity category, duration of follow-up, outcome definitions, number and type of cardiovascular events, and reported effect estimates with 95% confidence intervals. Priority was given to intention-to-treat estimates and adjudicated outcomes. First and recurrent events were recorded separately when available, and analyses were identified as prespecified, secondary, exploratory, or post hoc. For trials in which the entire population did not have both T2D and CKD, only estimates from the eligible T2D-CKD subgroup were extracted whenever separately reported. The principal outcomes were MI, stroke, and HHF, while MACE, cardiovascular death, urgent HF visits, and relevant cardiovascular composites were treated as supporting outcomes.

Risk-of-Bias Assessment

Two reviewers independently assessed risk of bias using the Cochrane Risk of Bias 2 (RoB 2) tool [7] for randomized trials. Judgements addressed bias arising from the randomization process, deviations from intended interventions, missing outcome data, measurement of outcomes, and selection of the reported result. Because RoB 2 is result-specific, the assessment focused on the cardiovascular estimates used in this review and considered whether each estimate arose from the parent trial, a prespecified subgroup analysis, or a post hoc analysis. Each domain and overall result were classified as low risk, some concerns, or high risk of bias. Disagreements were resolved through consensus or third-reviewer adjudication.

Data Synthesis

A structured narrative synthesis was undertaken because no trial directly randomized participants between different degrees of SGLT2 selectivity, and substantial heterogeneity was present in CKD definitions, baseline cardiovascular risk, follow-up duration, outcome definitions, and availability of CKD-specific estimates. Trials were organized along a prespecified selectivity framework comprising highly selective SGLT2 inhibition, intermediate or relatively lower SGLT2 selectivity with canagliflozin, and dual SGLT1/2 inhibition with sotagliflozin. Findings were compared according to HF-related outcomes, atherothrombotic outcomes, and cardiovascular mortality, with particular attention to eGFR, albuminuria, established ASCVD, and previous HF. Hazard ratios and 95% confidence intervals were presented as reported, but no unadjusted indirect comparison or pooled estimate across selectivity categories was interpreted as evidence of comparative superiority. Differences between agents were therefore treated as hypothesis-generating patterns that could reflect pharmacological selectivity, population characteristics, outcome reporting, or statistical power.

Results

Study Selection Process

The database search identified 674 records, comprising 210 from MEDLINE through PubMed, 245 from Embase, 89 from Cochrane CENTRAL, and 130 from the Web of Science Core Collection. After removal of 56 duplicates, 618 records underwent title and abstract screening, of which 412 were excluded. Full-text retrieval was sought for 206 reports, but 23 could not be obtained, leaving 183 reports for eligibility assessment. A further 167 reports were excluded because of an ineligible study design, publication type, population, intervention or comparator, absence of relevant cardiovascular outcomes, or lack of separately extractable T2D-CKD subgroup data. Ultimately, seven unique parent randomized trials, represented by 16 parent and linked secondary reports, were included in the systematic review (Figure 1).

**Figure 1:**
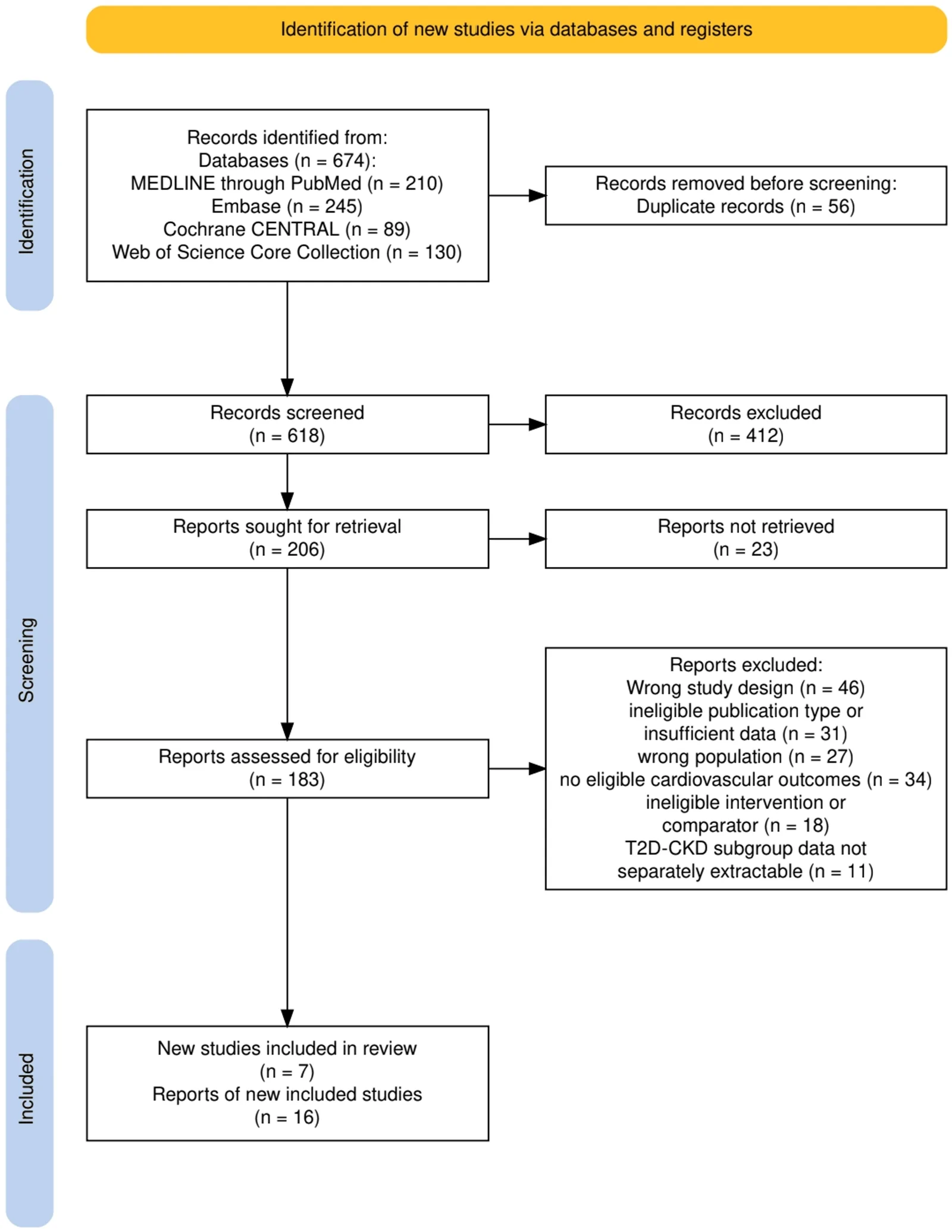
PRISMA flow diagram represents the study selection process. PRISMA, Preferred Reporting Items for Systematic Reviews and Meta-Analyses; T2D, type 2 diabetes; CKD, chronic kidney disease

Characteristics of the Selected Studies

Seven parent randomized controlled trials, represented by 16 parent and linked secondary reports, were included. Collectively, the trials enrolled 61,857 participants, although the eligible T2D-CKD population comprised either the complete cohort or an extractable subgroup. The interventions covered highly selective SGLT2 inhibitors, intermediate-selectivity canagliflozin, and dual SGLT1/2 inhibition with sotagliflozin, all compared with placebo and standard care. Follow-up ranged from 16 months to 4.2 years, with substantial variation in CKD definitions, albuminuria requirements, and baseline ASCVD and HF burden. The reported outcomes included MACE, MI, stroke, HHF, cardiovascular death, and related composites (Table 1).

**Table 1:** Characteristics and landmark cardiovascular findings of included trials. ASCVD, atherosclerotic cardiovascular disease; CKD, chronic kidney disease; CV, cardiovascular; CVD, cardiovascular disease; eGFR, estimated glomerular filtration rate; HF, heart failure; HHF, hospitalization for heart failure; HR, hazard ratio; MACE, major adverse cardiovascular events; MI, myocardial infarction; T2D, type 2 diabetes; UACR, urinary albumin-to-creatinine ratio; CI, confidence interval; DKD, diabetic kidney disease; SGLT, sodium-glucose cotransporter

Parent trial and contributing reports	Population and CKD definition	Intervention vs. comparator	Follow-up	Baseline cardiovascular profile	Landmark cardiovascular findings, HR (95% CI)
SCORED (Bhatt et al. [8]; MACE analysis: Aggarwal et al. [9])	10,584 adults with T2D and CKD; eGFR 25-60 mL/min/1.73 m²; albuminuria not required	Sotagliflozin 200-400 mg vs. placebo; dual SGLT1/2 inhibitor	Median 16 months	Established CVD: 48.6%; previous HF: 31.0%; median eGFR: 44.4	Total CV death, HHF, or urgent HF visits: 0.74 (0.63-0.88); total MACE: 0.77 (0.65-0.91); MI: 0.68 (0.52-0.89); stroke: 0.66 (0.48-0.91); CV death: 0.90 (0.73-1.12)
EMPA-REG OUTCOME (Zinman et al. [10]; CKD analysis: Wanner et al. [11])	7,020 adults with T2D and established CVD; CKD subgroup: 2,250 with eGFR <60 and/or UACR >300 mg/g	Empagliflozin 10 or 25 mg vs. placebo; highly selective SGLT2 inhibitor	Median 3.1 years	All participants had established CVD	In the CKD subgroup, CV death: 0.71 (0.52-0.98); HHF: 0.61 (0.42-0.87). Separate CKD-specific MI and stroke estimates were not reported
DECLARE-TIMI 58 (Wiviott et al. [12]; CKD-marker analysis: Zelniker et al. [13])	17,160 adults with T2D; 5,884 had ≥1 CKD marker, defined as eGFR <60 and/or UACR ≥30 mg/g; 548 had both markers	Dapagliflozin 10 mg vs. placebo; highly selective SGLT2 inhibitor	Median 4.2 years	Established ASCVD or multiple cardiovascular risk factors	CV death/HHF: 0.87 (0.72-1.05) with one CKD marker and 0.58 (0.36-0.94) with both markers; absolute risk reductions: 1.0% and 8.3%, respectively. The MACE effect was consistent across kidney-risk groups (interaction P = 0.64); separate CKD-specific MI and stroke estimates were not reported
VERTIS CV (Cannon et al. [14]; DKD-phenotype analysis: Cherney et al. [15])	8,246 adults with T2D and ASCVD; 4,114 classified by DKD phenotype using eGFR <60 and/or UACR ≥30 mg/g	Ertugliflozin 5 or 15 mg vs. placebo; highly selective SGLT2 inhibitor	Mean 3.5 years	All participants had established ASCVD; previous HF: 23.7% overall	HHF: 0.72 (0.31-1.69) in nonalbuminuric stage 3 CKD; 0.45 (0.28-0.73) in albuminuric stage 3 CKD; and 0.64 (0.42-0.97) in albuminuric stages 1-2 CKD (interaction P = 0.046). CKD-specific MACE, MI, and stroke estimates were not reported
DAPA-CKD (Heerspink et al. [16]; T2D analysis: Wheeler et al. [17])	4,304 adults with CKD; 2,906 had T2D; eGFR 25-75 and UACR 200-5,000 mg/g	Dapagliflozin 10 mg vs. placebo; highly selective SGLT2 inhibitor	Median 2.4 years	Established CVD: approximately 37%; previous HF: approximately 11% overall	In participants with T2D, CV death/HHF: 0.70 (0.53-0.92); all-cause mortality: 0.74 (0.56-0.98). Separate T2D-specific MACE, MI, stroke, and HHF estimates were not reported
CREDENCE (Perkovic et al. [18]; CV analysis: Mahaffey et al. [19]; stroke analysis: Zhou et al. [20])	4,401 adults with T2D and albuminuric CKD; eGFR 30-<90 and UACR >300-5,000 mg/g; all receiving renin-angiotensin-system blockade	Canagliflozin 100 mg vs. placebo; intermediate/relatively low SGLT2 selectivity	Median 2.62 years	Established CVD: 50.5%; previous HF: 14.8%	MACE: 0.80 (0.67-0.95); nonfatal MI: 0.81 (0.59-1.10); fatal/nonfatal stroke: 0.77 (0.55-1.08); HHF: 0.61 (0.47-0.80); CV death: 0.78 (0.61-1.00)
CANVAS Program (Neal et al. [21]; CKD analysis: Neuen et al. [22]; stroke analysis: Zhou et al. [23])	10,142 adults with T2D and high cardiovascular risk; CKD subgroup: 2,039 with eGFR <60 mL/min/1.73 m²; albuminuria not required	Canagliflozin 100-300 mg vs. placebo; intermediate/relatively low SGLT2 selectivity	Mean 188.2 weeks (approximately 3.6 years)	Established CVD in 71.6% of the CKD subgroup; previous HF in 14.4% overall	In the CKD subgroup, MACE: 0.70 (0.55-0.90). The stroke effect varied by baseline eGFR (interaction P = 0.01), with larger estimated protection at lower eGFR. Separate CKD-specific MI and HHF estimates were not reported

Risk of Bias Assessment

Using Cochrane RoB 2, all trials were judged to have low risk of bias in randomization, deviations from intended interventions, missing outcome data, and outcome measurement. DECLARE-TIMI 58 and DAPA-CKD were rated as having low overall risk, while SCORED, EMPA-REG OUTCOME, VERTIS CV, and the CANVAS Program raised some concerns because relevant estimates originated partly from secondary, exploratory, or post hoc analyses. CREDENCE had low risk for its prespecified MACE, MI, and HHF outcomes but some concerns for the post hoc stroke analysis. No trial was considered at high overall risk of bias (Table 2).

**Table 2:** Risk-of-bias assessment using Cochrane RoB 2. Risk of bias was assessed for the effect of assignment to intervention on the cardiovascular outcomes included in this review. Domain-level and overall judgements were classified as low risk, some concerns, or high risk of bias. Because RoB 2 is result-specific, the overall judgement could differ by outcome, as demonstrated by the low-risk assessment for the principal cardiovascular outcomes in CREDENCE and the judgement of some concerns for its post hoc stroke analysis [18-20]. Parent and linked secondary reports from the same randomized trial were assessed together, while the prespecified, secondary, exploratory, or post hoc status of each relevant analysis was considered when judging selection of the reported result. CKD, chronic kidney disease; CV, cardiovascular; DKD, diabetic kidney disease; HHF, hospitalization for heart failure; MACE, major adverse cardiovascular events; MI, myocardial infarction; RoB 2, Cochrane Risk of Bias 2; T2D, type 2 diabetes

Trial	Randomization process	Deviations from intended interventions	Missing outcome data	Outcome measurement	Selection of reported result	Overall risk	Basis for judgment
SCORED (Bhatt et al. [8]; MACE analysis: Aggarwal et al. [9])	Low	Low	Low	Low	Some concerns	Some concerns	Double-blind randomized design with adjudicated outcomes. However, the trial ended early because of funding loss; its primary endpoint was modified while investigators remained blinded, and the separate total MI and stroke analyses were post hoc
EMPA-REG OUTCOME (Zinman et al. [10]; CKD analysis: Wanner et al. [11])	Low	Low	Low	Low	Some concerns	Some concerns	Randomization, blinding, follow-up, and outcome adjudication were appropriate. The CKD-specific cardiovascular estimates used in this review originated from a post hoc subgroup analysis.
DECLARE-TIMI 58 (Wiviott et al. [12]; CKD-marker analysis: Zelniker et al. [13])	Low	Low	Low	Low	Low	Low	Large double-blind trial with adjudicated outcomes and intention-to-treat analysis. The kidney-function and albuminuria analysis was prespecified, although subgroup estimates were imprecise and not adjusted for multiplicity.
VERTIS CV (Cannon et al. [14]; DKD-phenotype analysis: Cherney et al. [15])	Low	Low	Low	Low	Some concerns	Some concerns	The parent trial was methodologically robust, but the DKD-phenotype analysis was post hoc, with multiple subgroup comparisons and no multiplicity adjustment.
DAPA-CKD (Heerspink et al. [16]; T2D analysis: Wheeler et al. [17])	Low	Low	Low	Low	Low	Low	Double-blind randomized design, high follow-up completeness, blinded outcome adjudication, and intention-to-treat analysis. Cardiovascular outcomes according to diabetes status were evaluated in a prespecified analysis.
CREDENCE (Perkovic et al. [18]; CV analysis: Mahaffey et al. [19]; stroke analysis: Zhou et al. [20])	Low	Low	Low	Low	Some concerns	Low for MACE, MI, and HHF; some concerns for stroke	The main cardiovascular outcomes were prespecified and centrally adjudicated. The detailed stroke analysis was post hoc, increasing concern about selective analysis and multiplicity for that outcome.
CANVAS Program (Neal et al. [21]; CKD analysis: Neuen et al. [22]; stroke analysis: Zhou et al. [23])	Low	Low	Low	Low	Some concerns	Some concerns	The parent trials had adequate randomization, blinding, follow-up, and adjudication. However, the CKD-specific estimates and renal-function interaction for stroke were derived from secondary or exploratory subgroup analyses.

Discussion

Principal Findings and Central Interpretation

Across the seven parent trials, protection against HF-related outcomes was the most consistent cardiovascular finding, extending across highly selective SGLT2 inhibitors, canagliflozin, and dual SGLT1/2 inhibition with sotagliflozin. Evidence for the prevention of atherothrombotic events was less consistent. The SCORED secondary analysis by Aggarwal et al. provided the clearest signal of concurrent reductions in myocardial infarction and stroke [9]. In contrast, CREDENCE and the CANVAS Program demonstrated MACE benefit with canagliflozin but did not establish statistically conclusive reductions in both individual outcomes [19-22]. Trials of highly selective SGLT2 inhibitors predominantly demonstrated reductions in HHF, cardiovascular death, or related composite outcomes, while CKD-specific estimates for myocardial infarction and stroke were frequently unavailable or inconclusive [11,13,15,17]. These findings raise the possibility that the cardiovascular effects of SGLT inhibition are not distributed uniformly between HF-related and atherothrombotic outcomes. However, because receptor selectivity was not randomized and the trials differed in population characteristics, kidney phenotype, outcome definitions, follow-up, and statistical power, the observed differences cannot be attributed confidently to pharmacological selectivity alone.

Shared and Robust HF Benefit

The reproducibility of HF-related benefit across the pharmacological spectrum suggests that it represents the most firmly established cardiovascular effect of SGLT inhibition [8,10,12,14,16,18,21]. Several complementary mechanisms may contribute, including natriuresis, osmotic diuresis, reduced interstitial congestion, restoration of tubuloglomerular feedback, improved ventricular loading conditions, and preservation of kidney function, with possible additional contributions from altered myocardial energetics and inflammatory signaling. The relatively early separation of HF event curves in several trial programs may be more compatible with prompt hemodynamic and cardiorenal effects than with gradual modification of atherosclerotic plaque biology, although clinical outcome data cannot determine the relative contribution of these mechanisms. The consistency of this HF signal provides a useful interpretive benchmark across trials. A reproducible reduction in myocardial infarction or stroke would therefore represent a potential extension beyond the best-supported class effect rather than another manifestation of protection against congestion and HF hospitalization.

Does Dual SGLT1/2 Inhibition Broaden Cardiovascular Protection?

The later analysis of SCORED by Aggarwal et al., in which MACE was a prespecified secondary outcome and total myocardial infarction and stroke were evaluated as individual post hoc outcomes, provides the most notable trial-derived signal that dual SGLT1/2 inhibition may extend cardiovascular protection beyond HF-related events. Sotagliflozin was associated with reductions in total MACE, myocardial infarction, and stroke [9]. Comparable CKD-specific reductions in both myocardial infarction and stroke were not consistently demonstrated across trials of highly selective SGLT2 inhibitors, although incomplete outcome reporting and limited statistical power constrain this comparison [11,13,15,17]. Partial inhibition of intestinal SGLT1 could plausibly attenuate postprandial glucose excursions and glycemic variability, while potential effects on endothelial dysfunction, platelet activation, inflammation, oxidative stress, or SGLT1-expressing cardiovascular tissues remain biologically plausible but insufficiently established. Importantly, SCORED compared sotagliflozin with placebo rather than an active selective SGLT2 inhibitor [8,9]. Its findings therefore support a testable mechanistic hypothesis but do not demonstrate the superiority of dual inhibition or establish SGLT1 engagement as the cause of the observed atherothrombotic benefit.

Canagliflozin as an Intermediate Pharmacological Phenotype

Canagliflozin provides a useful conceptual bridge between highly selective SGLT2 inhibition and dual SGLT1/2 inhibition because its relative selectivity for SGLT2 is lower than that of empagliflozin, dapagliflozin, and ertugliflozin, while remaining distinct from the more balanced SGLT1/2 activity of sotagliflozin. Across CREDENCE and the CANVAS Program, canagliflozin reduced HHF and MACEs, whereas individual myocardial infarction and stroke estimates were generally directionally favorable but statistically inconclusive [19-21]. The CANVAS analysis by Neuen et al., together with the exploratory stroke analysis by Zhou et al., also raised the possibility that cardiovascular effects may vary according to baseline kidney function, although these subgroup findings require cautious interpretation [22,23]. Collectively, the evidence suggests a potentially informative pattern in which highly selective agents predominantly demonstrate cardiorenal and HF-related protection, canagliflozin occupies an intermediate outcome profile, and sotagliflozin provides the strongest current signal for combined HF and atherothrombotic benefit [9,11,13,15,17,19,22]. This pattern should not be interpreted as a pharmacological dose-response relationship because receptor selectivity is continuous, depends on drug concentration and tissue exposure, and was not randomized across the included trials.

CKD Phenotype as a Modifier of Cardiovascular Treatment Value

The findings support interpreting chronic kidney disease as a multidimensional cardiovascular phenotype rather than a binary eligibility criterion. Reduced eGFR and increased albuminuria represent partly independent dimensions of kidney disease that identify overlapping but potentially different pathways of cardiovascular vulnerability. Declining kidney function is associated with vascular calcification, inflammation, anemia, arrhythmia, and competing mortality, whereas albuminuria reflects glomerular injury and may also indicate systemic endothelial dysfunction. In the DECLARE-TIMI 58 analysis by Zelniker et al., participants with both reduced kidney function and albuminuria experienced the greatest absolute reduction in cardiovascular death or HHF [13]. The VERTIS CV analysis by Cherney et al. similarly suggested greater protection against HF hospitalization in albuminuric or more advanced diabetic kidney disease phenotypes [15]. The CANVAS Program generated an exploratory interaction between kidney function and stroke benefit [23], while SCORED demonstrated cardiovascular protection in a population with substantial variation in albuminuria [8]. Although these subgroup findings are not uniformly definitive, they suggest that kidney phenotype may influence both the underlying balance of HF-related and atherothrombotic risk and the absolute benefit achievable with treatment. Clinical interpretation may therefore be improved by considering kidney function and albuminuria jointly rather than treating the presence of CKD alone as a sufficiently informative risk category.

Alternative Explanations for the Apparent Selectivity Gradient

The apparent gradient in cardiovascular outcomes must be interpreted within the substantial clinical and methodological differences among the included trials. The prevalence of established ASCVD, baseline HF burden, kidney function, albuminuria, prevention setting, follow-up duration, background cardiorenal therapy, and outcome definitions varied considerably [8,10,12,14,16,18,21]. Analytic approaches also differed, particularly in their use of first versus total events and in the statistical power available for individual myocardial infarction and stroke outcomes. SCORED enrolled an entirely T2D and CKD population and subsequently provided dedicated analyses of total MACE, myocardial infarction, and stroke [8,9]. In contrast, several selective SGLT2 trials contributed only CKD subgroup estimates or composite cardiovascular outcomes, with separate CKD-specific myocardial infarction and stroke results unavailable [11,13,15,17]. SCORED was also terminated prematurely because of funding loss, which shortened follow-up, although termination was not prompted by an observed treatment effect [8]. Consequently, the stronger atherothrombotic signal associated with sotagliflozin may partly reflect differences in baseline risk, endpoint ascertainment, analytic strategy, reporting completeness, or precision. These factors do not exclude a possible role for receptor selectivity, but they prevent the observed cross-trial pattern from being interpreted as direct evidence of a biological gradient.

Clinical Implications and Outcome-Oriented Treatment Selection

The findings support an outcome-oriented clinical framework rather than a hierarchical ranking of individual agents. When prevention of HF hospitalization, recurrent congestion, or cardiorenal instability is the predominant concern, the consistency of benefit supports the use of an SGLT inhibitor with an established indication across the pharmacological spectrum [8,11,13,15,17,19,21]. Among patients with CKD and substantial residual atherothrombotic risk, sotagliflozin may be of particular interest because of the reductions in myocardial infarction and stroke observed in SCORED, although these findings do not establish superiority over selective SGLT2 inhibitors [9]. Canagliflozin has strong evidence for reducing cardiorenal events and MACE in albuminuric diabetic CKD [18-20], while highly selective SGLT2 inhibitors retain substantial evidence across HF, cardiovascular, and kidney outcomes [10-17]. Treatment selection should therefore integrate the patient’s dominant cardiovascular phenotype with kidney function, albuminuria, regulatory indication, tolerability, volume status, ketoacidosis risk, genital infections, gastrointestinal adverse effects, limb history, cost, and local availability. This approach recognizes that residual cardiovascular risk may be predominantly HF related, atherothrombotic, or mixed, while preserving individualized clinical judgement and avoiding conclusions unsupported by direct comparative evidence.

Limitations, Research Priorities, and Final Perspective

This review is limited by the absence of direct randomized comparisons between selective SGLT2 inhibitors and sotagliflozin, the small number of parent trials, considerable clinical heterogeneity, and reliance on subgroup or post hoc analyses for several CKD-specific outcomes [9,11,13,15,17,20,22,23]. Incomplete reporting of separate myocardial infarction and stroke estimates created an uneven evidence base, while cross-trial comparisons introduced a risk of ecological inference and limited the ability to distinguish pharmacological effects from differences in the enrolled populations. Future research should prioritize adequately powered head-to-head trials or individual-participant-data analyses using standardized definitions of myocardial infarction, stroke, HHF, and cardiovascular death. Consistent stratification by kidney function, albuminuria, atherosclerotic cardiovascular disease, and HF phenotype, together with formal interaction testing between receptor selectivity and outcome type, would improve comparability. Analyses of absolute benefit, recurrent events, competing risks, and adverse outcomes would further enhance clinical applicability, while mechanistic studies are needed to determine whether SGLT1 inhibition meaningfully influences atherothrombosis. The available evidence supports HF protection as the most consistent cardiovascular consequence of SGLT inhibition and raises a clinically relevant hypothesis that greater SGLT1 engagement may broaden protection toward myocardial infarction and stroke [9]. This hypothesis remains unproven and should be regarded as a focused priority for comparative and mechanistic investigation.

## Conclusions

SGLT inhibition in adults with T2D and CKD provides consistent protection against heart failure-related events, while its effects on myocardial infarction and stroke appear less uniform. Sotagliflozin provides the strongest current signal for combined heart failure and atherothrombotic protection, whereas canagliflozin may occupy an intermediate outcome profile, and highly selective SGLT2 inhibitors remain supported predominantly by robust cardiorenal and heart failure evidence. These differences are clinically relevant but cannot yet be attributed to receptor selectivity because they arise from indirect comparisons across heterogeneous trials. The principal take-home message is that SGLT inhibitors should not be assumed to have identical cardiovascular outcome profiles solely because they share a therapeutic class. Treatment selection should instead consider the patient’s dominant residual risk phenotype, whether heart failure-related, atherothrombotic, or mixed, while future direct comparative research determines whether broader SGLT1 engagement extends cardiovascular protection beyond the established heart failure benefit.
